# Impact of Glass Free Volume on Femtosecond Laser-Written Nanograting Formation in Silica Glass

**DOI:** 10.3390/ma17020502

**Published:** 2024-01-20

**Authors:** Nadezhda Shchedrina, Maxime Cavillon, Julien Ari, Nadège Ollier, Matthieu Lancry

**Affiliations:** 1 Institut de Chimie Moléculaire et des Matériaux d’Orsay, Université Paris-Saclay, Avenue des Sciences, 91400 Orsay, France; maxime.cavillon@universite-paris-saclay.fr (M.C.); julien.ari@universite-paris-saclay.fr (J.A.); 2 Laboratoire des Solides Irradiés, École Polytechnique-CEA-CNRS, 91128 Palaiseau Cedex, France; nadege.ollier@polytechnique.edu

**Keywords:** fs-laser, type II modifications, nanogratings, silica glass, densified glass, free volume

## Abstract

In this study, we investigate the effects of densification through high pressure and temperature (up to 5 GPa, 1000 °C) in the making of nanogratings in pure silica glass, inscribed with femtosecond laser. The latter were monitored through retardance measurements using polarized optical microscopy, and their internal structure was observed under scanning electron microscopy. We reveal the difficulty in making nanogratings in densified silica glasses. Based on this observation, we propose that free volume may be a key precursor to initiate nanograting formation.

## 1. Introduction

The variety of femtosecond (fs) laser-induced modifications in glass paves the way for a multitude of structural alterations initiated through the nonlinear absorption of laser pulse energy. The nature of these modifications in optical glasses varies based on the laser-writing parameters, and they are classified in the literature into three main types [1]. Type I modifications can be observed as smooth and homogeneous changes in the refractive index at lower energy. Early studies demonstrated the potential of fs-lasers to induce 3D refractive index profiling in silica glass, a foundational step in creating waveguides [2]. Type II modifications are distinguished by an anisotropic change in the refractive index. Under specific pulse duration, frequency, and energy conditions, a strong form birefringence appears, which originates from periodic lamellar nanostructures oriented perpendicular to the laser polarization [3]. At higher laser intensities, Type III modifications occur, marked by the formation of nano/micro-voids with a densified shell due to localized micro-explosions.

Of particular interest are Type II modifications, associated with the formation of nanogratings. Fs-laser-induced nanogratings find expansive applications in several technological domains. They play a central role in the creation of long-term optical data storage devices [4,5], thermal optical sensors [6,7], and microfluidics [8,9]. Importantly, they are also employed in the fabrication of various optical elements, including waveguides, light polarization converters [10,11], and other birefringent elements [12]. Despite the broad scope of their applications, a comprehensive understanding of the mechanisms behind nanograting formation in glass remains to be achieved. This is crucial, as it impacts their fabrication and, consequently, the optimization of their use in various technological contexts.

Central to the nanograting formation process is the phenomenon of multiphoton ionization, wherein photon absorption facilitates energy transfer from the incident light to the solid glass structure [13]. As the laser intensity exceeds specific thresholds, it results in the generation of plasma, characterized by a high-density free-electron cloud [14]. Interference between the incident laser light and scattered light from the inhomogeneities in the glass matrix results in periodic modulations in the electron plasma concentration, leading to nanostructural changes in the glass [15]. Simultaneously, plasma hotspots evolve into elongated nanoplasma regions because of local field enhancement, which occurs perpendicular to the polarization [16]. These nanoplasma regions are “forced-arranged” by light into distinct periodic patterns, oriented in a direction orthogonal to the laser’s polarization vector. Finally, plasma-mediated oxide decomposition occurs, resulting in nanolayers made of an assembly of oblate nanopores [17,18].

While there is an understanding of the mechanisms behind nanograting formation, determining the exact precursors in glass remains a debated topic in the field of laser–matter interactions. One hypothesis [19] posits that the inception of nanogratings does not have any pre-existing precursors. Instead, modeling studies suggest that the initial laser pulse generates nanopores or nanovoids, which subsequently influence the light organization [15,20]. An alternative theory [21] emphasizes the role of point defects and color centers as the initial precursors for nanograting formation. These defects are hypothesized to be either intrinsic, pre-existing within the material, or extrinsically induced by the initial laser pulse (e.g., STHs [22]).

Building upon the existing theories of nanograting formation, our current research introduces a hypothesis that emphasizes the significance of free volume in the nanograting seeding process. Free volume in glass refers to sub-nanometer-scale voids or spaces within its amorphous structure [23,24,25,26]. In silica glass, this free volume is significant given the inherent arrangement of silicon and oxygen atoms, which creates a 3D network of n-membered rings with notable interstitial spaces [27]. The density of silica glass inversely correlates with its free volume; as the glass becomes denser through processes like high-pressure, high-temperature (HPHT) treatment, its free volume decreases [25]. In silica glass, its density or specific volume can be reduced by up to 22% under high pressure (HP) [28], highlighting its intrinsic porous nature at the sub-nanometer scale and its high initial free volume. This perspective could explain why materials like silica [14] and germanium dioxide [29], which inherently possess large free volumes in their networks, demonstrate a pronounced ability to form nanogratings compared with most other glass [30].

This paper presents, for the first time, an investigation of nanograting writing in pristine silica glass compared with densified silica glass subjected to high-pressure, high-temperature (HPHT) conditions. Prior studies have extensively explored the properties of densified glass through techniques such as Raman spectroscopy [31], X-ray diffraction [32], Brillouin scattering [33], and positron annihilation spectroscopy (PAS) [25], which have demonstrated a reduction in glass free volume indicators such as void size and rings statistics. By utilizing fs-laser writing in four different silica glass samples, each with a distinct density, and varying both pulse energy and pulse density followed by birefringence measurements, we strive to gain insights into how density and inherent interstitial voids affect the dynamic of nanograting formation. This exploration contributes to our broader understanding of the mechanisms behind nanograting creation and their potential optimization across various applications.

## 2. Materials and Methods

**Figure 1:**
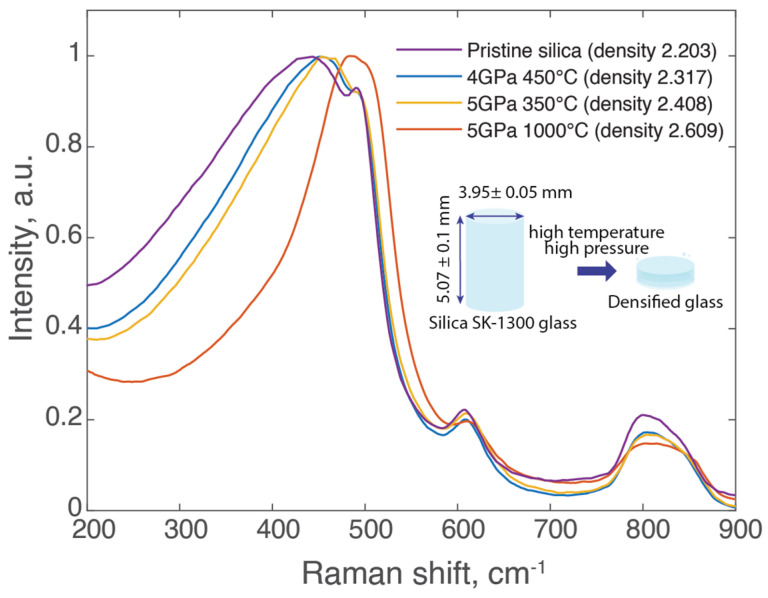
Raman scattering spectra of silica glass samples alongside a schematic representation of the densification process.

The material used for this study was Synthetic Fused Silica SK-1300 glass (OHARA GMbH, Hofheim, Germany), fabricated with the vapor axial deposition process (OH < 200 ppm). The silica samples used in our experiments were cylindrical in shape, with a diameter of 3.95 mm and a thickness of approximately 3 mm. A schematic representation of these samples is included in the insert of Figure 1. Densification of the silica glass was achieved using a high-temperature, high-pressure belt press [34]. The selected pressure and temperature conditions were chosen based on the literature [34] to provide a range of densities from 2.2 to 2.6, with the highest one being close to α-quartz density. Consequently, four samples were prepared: pristine silica and three densified samples (4 GPa at 450 °C, 5 GPa at 350 °C, and 5 GPa at 1000 °C); the densities of these samples were determined to be 2.203 ± 0.001, 2.317 ± 0.007, 2.408 ± 0.013, and 2.609 ± 0.006, respectively, where the error represents the standard deviation from three separate measurements. The density measurements were conducted using the Archimedean sink–float method, measuring the samples’ weights in air (*m_a_*) and when submerged in toluene (*m_l_*). We employed the equation *d* = (*m_a_* × *ρ_toluene_*)/(*m_a_* − *m_l_*) to calculate the density, where *ρ_toluene_* is the density of toluene, determined by its temperature-dependent equation (density (T) = 0.8845 − 0.9159 × 10^−3^ × T + 0.368 × 10^−6^ × T_2_, T in °C) [35]. Considering the optical properties of densified silica, it has been observed that the refractive index generally scales up with increased density in silica glass [36]. As for the optical bandgap, only minor changes (typically less than 5–10%) can be observed in densified silica. This can be inferred from calculations conducted on crystalline polymorphs of silica [37].

This study employed an fs-laser system (Satsuma, Amplitude Systemes Ltd., Pessac, France) operated at 1030 nm, with a repetition rate of 10–100 kHz and a pulse duration of around 300 fs. The chosen repetition rate (10–100 kHz) was low enough to avoid any pulse-to-pulse heat accumulation effects. The laser beam was focused 200 μm beneath the sample surface using a 0.6 NA aspheric lens. The laser-writing process was conducted at a scanning speed of 0.1 mm/s, with energy ranging from 0.05 μJ to 2 μJ. In a second set of experiments, the scanning speed was decreased from 5 to 0.005 mm/s to achieve a range of pulse density between 2 to 50,000 pulses/µm. The pulse density was calculated from the scanning speed and the repetition rate using the formula *N = f/v,* where *N* is the number of pulses per micron, *f* is the repetition rate in kHz, and *v* is the scanning speed in mm/s. During the laser-writing procedure, the sample was moved along the X-axis. The laser light was linearly polarized and set in two orientations (writing configurations) parallel (Xx) and perpendicular (Xy) to the laser-writing direction.

**Figure 2:**
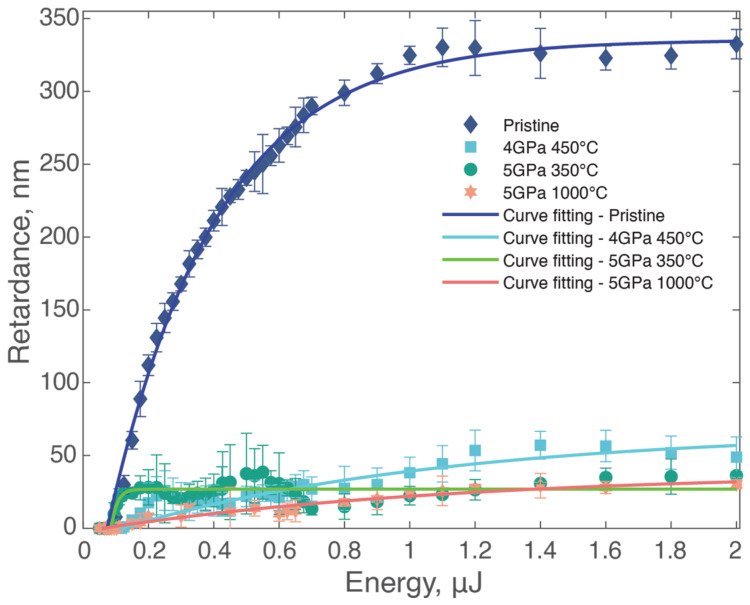
Mean retardance of laser-written structures plotted against pulse energy. Experimental conditions: λ = 1030 nm; τ = 250 fs; f = 100 kHz; v = 100 μm/s, resulting in a pulse density of 1000 pulses/µm and energy, E, varying from 0.05 to 2 μJ.

**Figure 3:**
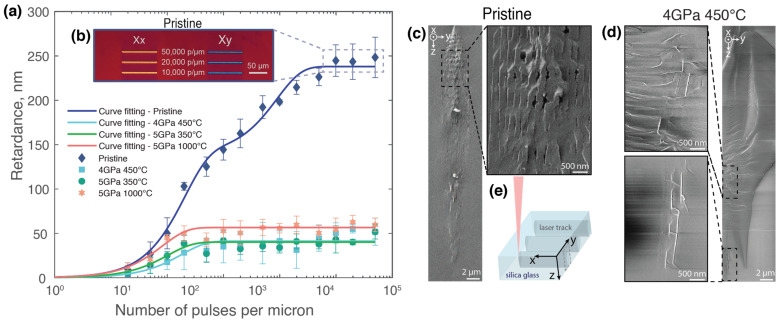
(**a**) Mean retardance of laser-written structures plotted against a number of pulses per micron. Experimental conditions: λ = 1030 nm, τ = 350 fs, E = 1 μJ, f = 10–100 kHz, and v = 0.002–5 mm/s for a pulse density from 2 to 50,000 pulses/µm. (**b**) Optical microscope image of laser-written structures using a crossed polarizer and analyzer and a full retardation waveplate inserted at 45°, indicating the orientation of the slow axis for pristine sample. (**c**) SEM images of a cross-section of the laser track (1000 pulses/µm, 1 μJ, Xy writing configuration) in the pristine sample and (**d**) in the 4 GPa 450 °C sample. (**e**) Scheme of the sample orientation for the SEM analyses.

The formed optical modifications were examined using an Olympus BX51 polarized optical microscope (Olympus Corporation, Tokyo, Japan) in transmission mode. To measure the optical retardance, which is proportional to the linear birefringence, induced by fs-laser direct writing, we employed the Sénarmont compensator technique [38]. A quarter-waveplate is oriented at 45° to the axis of the linearly polarized light entering the microscope. As light passes through the birefringent sample, it undergoes a phase shift, resulting in elliptically polarized light. The rotating analyzer is then adjusted to achieve extinction, and its angle, *θ,* is rotated directly proportional to the retardance, following the relation *R* = (*λ* ∙ *θ*)/180, where *λ* is the probe wavelength in nm, and *R* is the relative retardance or optical path difference in nm. The measurements represent mean values, averaging from a series of three independent measurements. Data fitting was performed as a guide to the eye for Figure 2 and Figure 3, using an asymptotic exponential function:

(1) $$R=a\times \left(1-exp\left(-\frac{x}{b}\right)\right),$$

where *R* is the retardance, *x* represents the input variable (either pulse energy or number of pulses per micron), and *a* and *b* are the fitting parameters. Additionally, we used a full waveplate to determine the slow/fast axis orientation. This method also helps to visually confirm the dependence of the birefringence orientation with the writing laser’s polarization. 

To analyze the cross-sections of the laser tracks, cleaved samples were examined using a field emission gun scanning electron microscope (FEG-SEM, ZEISS SUPRA 55 VP, Zeiss, Oberkochen, Germany) to study their morphology. Prior to laser irradiation, Raman spectroscopy measurements were taken using an externally doubled diode laser from Spectra-Physics, operating at an excitation wavelength of 488 nm, with a 1200 L/mm grating, a slit width of 50 μm, and power of 28.5 mW. Spectra were normalized by the total integrated area.

## 3. Results

In this study, we employed a set of four silica glass samples, one of which was pristine, while the remaining three were subjected to densification via a belt press technique. The densification process, executed under varying conditions of temperature and pressure using the same HPHT method for all samples, reliably generated distinct densities. The variation in free volume can be indirectly assessed through molar volume, expressed as $\frac{M}{\rho }$ (where *M* is molar mass, and ρ is density). Applying this to our samples indicates a molar volume decrease of approximately 4.9%, 8.5%, and 15.6% for densified samples 4 GPa 450 °C, 5 Gpa 350 °C, and 5 Gpa 1000 °C, respectively, when compared with the pristine glass with a molar volume of 27.27 cm³/mol. Positron annihilation spectroscopy (PAS) studies have demonstrated that the HPHT densification process significantly impacts void size in the silica network, linearly reducing the average void volume from 65 Å³ to as small as 10 Å³ with a densification of 22% [25]. A schematic representation illustrating the densification process and the dimensions of the samples is provided in the insert of Figure 1.

The Raman spectra of the four samples were obtained and are presented in Figure 1. Notable differences were observed in the spectral shapes between the densified samples and the pristine sample. In our observations, the R-band at 440 cm^−1^ in the densified samples notably shifted toward higher frequencies, and concurrently, the full width at half maximum (FWHM) of this R-band was observed to decrease. These changes indicate reduced average Si-O-Si angles, consistent with the increased density of the silica glass [31]. The densification process compacts the structure of silica glass, causing silica tetrahedra to tilt closer to each other and reducing the available volume between the atoms. This results in a more uniform and, thus, narrower range of Si-O-Si bond angles. The uniformity in bond angles restricts the vibrational frequency range of the Si-O-Si bonds, which is reflected as a sharper and more defined Raman band in our spectral analysis. Variations in the intensities of the D_1_ and D_2_ Raman bands, which are linked to local density indicators, were also noted.

Two distinct experimental series were conducted to investigate nanograting formation. In the first series, the pulse count remained constant as the energy varied between 0.05 µJ and 2 µJ. The laser-writing parameters and this energy range were chosen based on prior research [39] and our focus on Type II fs-laser-induced structures, which are associated with nanograting formation and result in a permanent form birefringence. The second series maintained a constant energy while varying the pulse count from 2 to 50,000 pulses per micron. Subsequent optical retardance measurements were performed on the laser-written structures in each sample type. In the experiment with energy variation, Figure 2 illustrates the retardance values within the irradiated areas inside the laser tracks across differing pulse energies for the four types of samples.

The lowest observed energy yielding a non- or low-birefringent optical contrast marks the Type I threshold (if any). In addition, the orientation of the stress-induced birefringence within this Type I regime remains independent of the writing polarization orientation. In contrast, Type II corresponds to a non-zero birefringence (here, an optical retardance) whose slow/fast axis orientation is quasi-linearly dependent on writing laser polarization, thus indicating nanograting formation [40], which we initially detect using the full waveplate technique and further confirmed with SEM. To precisely determine this threshold, we incrementally increased energy in very small steps (ranging from 0.015 µJ to 0.075 µJ) at the lower energy range (from 0.05 µJ to 0.125 µJ). This approach ensured a precise determination of the threshold energy, beyond which, we increased the step size (0.025 µJ to 0.2 µJ) for practicality in measurements. For the pristine sample, the threshold energy was approximately 0.125 µJ. The retardance then rapidly increased to around 330 nm at 1 µJ, beyond which, it stabilized. Different degrees of densification in silica glass only subtly influence the threshold energy (around +/−0.02 µJ). However, they exhibited fluctuating but generally low retardance levels (typ. below 50 nm), with values notably below those of the pristine silica sample.

For the subsequent experiment, an energy of 1 µJ was selected, as this value represented a plateau in retardance for all samples. The focus was shifted to varying the number of pulses per micron, as previous studies have demonstrated that higher pulse densities lead to more pronounced nanograting formation with a lower period [15,41]. Here, the retardance within the irradiated areas in the pristine sample initially increased gradually, reaching a value of around 30 nm at 10 pulses per micron. It then exhibited a more pronounced increase, peaking at approximately 270 nm at 50,000 pulses per micron (Figure 3a). In contrast, the retardance values within the irradiated areas of the densified samples remained substantially lower, not surpassing 55 nm.

In both experimental conditions, indirect evidence of nanograting formation was observed in the pristine samples. In Figure 3b, an optical micrograph using a full waveplate displays the behavior of the pristine silica glass sample. The full waveplate adds a fixed optical path difference, which results in the emergence of interference-based colors [42]. These images reveal the neutral axes of the birefringence: the orange (Xx writing configuration) and blue (Xy writing configuration) colors correspond to the orientation of the birefringence’s fast and slow axes, respectively, relative to the laser polarization. In Figure 3b, the clear contrast between orange and blue serves as an indirect confirmation of nanograting formation since it demonstrates the formation of a birefringence whose orientation is polarization-dependent [3]. Indeed, the change in color directly correlates to the 90° rotation of the birefringence slow axis when the writing laser’s polarization is rotated by 90°. Contrastingly, no such evidence of nanograting formation was observed in the densified sample. In the densified samples, the laser tracks appear uniformly colored despite the writing configuration, signaling the absence of ordered nanogratings while stress-induced birefringence is still present.

To complement this view, SEM micrographs in Figure 3c,d provide a comparative cross-sectional analysis of the nanogratings in two silica glasses, respectively, 4 GPa 450 °C and the pristine glass. For the pristine sample (Figure 3c), images reveal an ordered array of nanolayers, consistent with expectations for laser tracks written with 1000 pulses per micron at an energy of 1 µJ in the Xy writing configuration. Here, the laser polarization is perpendicular to the laser-writing direction, resulting in nanogratings that are vertically aligned within the laser track. However, it should be noted that some tilt in the nanogratings can occur because of variations in the local material response [43]. In contrast, Figure 3d displays the cross-section in densified silica glass (4 GPa 450 °C), where the nanogratings’ periodicity appears quite disrupted. Only a few vertical nanolayers can be observed, which are visible in the magnified images of Figure 3c, highlighting the “negative effect” of densification on nanograting organization. Furthermore, horizontal striations are visible in the SEM micrographs (Figure 3d) along the cracks, which are typical fracture facies due to strain relaxation in cleaved samples.

**Figure 4:**
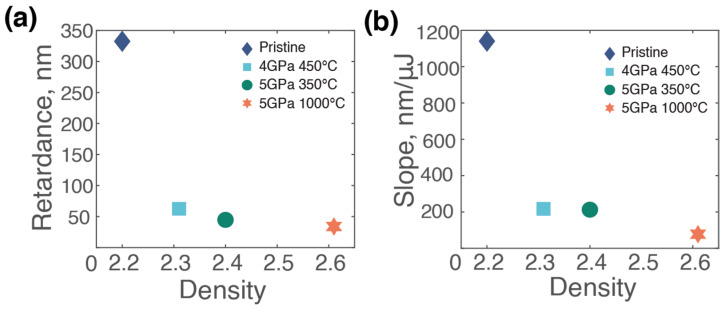
Analysis of retardance for the set of samples with varying energy: (**a**) plot of the maximum retardance for each sample against the density of the samples; (**b**) plot of the slope of the curve at the origin (up to 0.2 µJ) for each sample against the density of the samples.

Returning to the dependence of retardance on pulse energy, a more detailed analysis reveals correlations with the density of the studied samples. For instance, Figure 4a presents the dependency of the maximum retardance value on the density of the samples. For the pristine silica, this value is notably high (332 nm), while for the densified samples, it remains considerably lower, reaching a minimum (34 nm) for the most densely packed glass (5 GPa at 1000 °C). A similar trend is observed when examining the slope of the curve at the origin (up to 0.2 µJ), as shown in Figure 4b. The slope at the origin of the retardance kinetics curve in Figure 4b, measured just above the threshold energy, serves as an indicator of the material’s initial photosensitivity. This parameter reflects the efficiency of nanograting formation at the onset, revealing the relative ease of imprinting nanogratings in the glass according to its initial density.

## 4. Discussion

In this section, we explore the initial steps of nanograting formation, focusing on the role of glass density and underlying structure in silica glass.

According to the prevailing theory, nanograting formation initiates with dielectric constant inhomogeneities that serve as process precursors or seeds, and which also play a role in scattering centers in the glass matrix. Within this model [15,20], incident light interacts with scattering centers in the glass structure, leading to multiple scattered wave interferences. Within the interference pattern, these seeds, in turn, generate spherical nanoplasma hotspots because of increased plasma density. As the process evolves, the influence of light polarization reshapes this nanoplasma into an oblate shape [16]. For seeds with a lower dielectric constant compared with the surrounding material, the local field enhancement results in the maxima perpendicularly to light polarization, whereas it is the reverse for a higher dielectric constant [15,44]. Through the plasma-mediated nanocavitation process, nanopores are created that, over numerous laser pulses, eventually merge to form nanolayers [15]. Nanogratings in the glass consistently form perpendicular to light polarization.

The literature suggests various potential seeds, either native to the glass or induced by laser pulses. Modeling studies of bulk nanogratings are usually based on a low dielectric constant seed as an initial “nanovoid” [15,20]. Some studies have suggested point defects like E’; ODC; and, generally, color centers [21], as well as transient defects like self-trapped electrons (STEs) [18,45] and self-trapped holes (STHs) [22] as seeds. In discussing potential precursors, we must consider the necessity of a lower change in the refractive index to facilitate the plasma field enhancement perpendicularly to light polarization. Notably, most point defects, as well as STEs, are unlikely candidates, as they contribute to a positive change in the local refractive index. Another counterargument against the presumption of point defects acting as seeds comes from observations concerning HPHT densification, which increases the initial concentration of defects such as E’ centers and ODCs [46]. Nevertheless, as described above, we evidenced that the formation of nanogratings remains notably suppressed in highly densified glass, indicating a less efficient generation process. Recent studies have considered the potential role of STHs as seeds for nanograting formation [22]. However, certain intrinsic characteristics of STHs cast doubt on their feasibility as seeds. Primarily, the size of STHs, approximately 1 Å, makes them too small to function effectively as scattering centers. Moreover, some studies indicate a lower stability of STHs in neutron-irradiated or in densified silica, particularly at room temperature, compared with pristine silica [47]. Hypothetically, this should instead facilitate nanograting formation in densified silica because of the accumulation of more stable STHs between laser pulses.

Our research introduces a new hypothesis highlighting the role of free volume and inherently related voids as precursors for nanograting formation. This aligns with the observed ease of nanograting formation in silica, a glass material characterized by a very high free volume. The efficiency is even more pronounced in nanoporous silica forms like aerogels [48], which require fewer pulses to form high birefringence given their increased free volume. So, we suggest that the initial sub-nanometer or -nanoscale porosity (the so-called glass free volume) of the glass plays a role as a low-density (low dielectric constant) seed, initiating the scattered waves that lead to the plasma spatial organization, namely, an arrangement of regularly spaced hot plasma layers. Then, oxide decomposition occurs [17] within these hot plasma nanolayers, resulting in nanograting imprinting. According to current models [15], the concentration of these “nanopores” significantly influences the nanograting-seeding process, resulting in a shorter average period for the nanogratings. The pore size is also an important factor; numerical models have successfully used voids of up to 10 nm [15,20]. Experimentally, it has been observed that nanogratings are more easily imprinted in nanoporous sol–gel silica provided that nanopores are not too big [48,49].

Conversely, multicomponent glasses such as aluminoborosilicate glasses, with their denser networks and lower free volume, exhibit narrower processing windows for nanograting formation, partly attributable to their reduced free volume [39]. First one needs to consider that the chemical composition, particularly the addition of B and Al atoms in silicate glasses, leads to a less ”open” glass network, translating into a lower free volume, as indicated by a higher atomic packing density [50]. Secondly, the free volume model also offers a comprehensive framework for understanding the relationship between glass viscosity, *T*, and dependence, especially in the temperature range, from the glass transition temperature (*T_g_*) up to the melting temperature (*T_m_*) [51]:

(2) $$\eta =\eta_{0}\exp\left(\frac{BV_{0}}{V_{f}}\right),$$

where η is glass viscosity, *V_f_* is the free volume, *V*_0_ is the volume of a molecule, and *η_0_* and *B* are constants. According to this model, free volume, *V_f_*, can be represented by the equation:

(3) $$V_{f}=V\;–\;V_{0}=\frac{V_{0}\left(T\;–\;T_{0}\right)}{T_{0}},$$

where *V* is the total volume, and *T*_0_ is a critical temperature. From an experimental point of view, in silica-based glasses, *T*_0_ is typically much smaller compared with aluminoborosilicate glasses such as commercial varieties like B33, AF32, BK7, etc. This difference in *T*_0_ translates into a significantly lower-free-volume aluminoborosilicate, which can be directly correlated with its reduced ability to form nanogratings. Indeed, the processing window for nanograting formation has two boundaries: the lower energy bound is influenced by the glass’s free volume (seeding the process), while the upper limit, defined by the maximum energy beyond which no more nanogratings survive, is less directly but still related to free volume through the glass viscosity (*T*) and the laser heating–cooling profile. While nanoporous layers can be generated through plasma-mediated nanocavitation, at higher energies or repetition rates, these nanolayers can be erased by subsequent heat pulses within a few 10s of nanoseconds, as modeled in our recent studies [52].

We hypothesize that free volume and, thus, the resulting voids are primordial in determining both the plasma spatial organization and the nanocavitation process. As previously mentioned, varying density from 2.203 to 2.609 yields a molar volume reduction of up to 15.6%. The literature indicates that a 22% density increase can shrink the void size to below 10 Å³, a stark contrast to the 65 Å³ of pristine silica [25,41]. This agrees with the Raman spectroscopy results (Figure 1), which reveal notable shifts in the R-band toward higher frequencies in densified samples along with a decreased FWHM, indicating a reduction in average Si-O-Si angles, signifying increased glass density [31,47]. This compaction results in a narrower Si-O-Si bond angle range, as reflected in more defined Raman bands. The shift in the D_2_ peak toward higher frequencies further corroborates this densification [34].

In our investigations of silica with varying densities, we manipulated energy levels and pulse numbers to assess their impact on nanograting formation. Utilizing the maximum value and slope of the retardance curve as indicators, we found marked differences between pristine and densified samples. While the threshold energy remained quite consistent across all samples, densified samples exhibited significantly lower retardance values (Figure 2 and Figure 3a). In the results shown in Figure 2, the minimal differences in optical retardance between the densified samples, as compared with the more pronounced retardance in the pristine sample, can primarily be attributed to the high degree of densification across all samples. The degree of densification in this study is higher than the metamict phase threshold of silica, where further densification through any kind of irradiations becomes ineffective [53]. In contrast, more significant differences are expected in samples with densities ranging from 2.20 to 2.27, i.e., below the metamict phase density.

As illustrated in Figure 4, both maximum retardance and slope values decreased with increasing density. Contrary to the well-formed nanogratings in the pristine samples, the densified counterparts tend to avoid the generation of nanogratings, as evidenced by our SEM analysis (Figure 3c,d). The observed lower retardance could potentially stem from a reduced number of seeds, which would also imply fewer nanopores generated per unit volume. The background birefringence in the highest densified sample can be attributed to the stress-induced birefringence. Meanwhile, the reduced slope indicates a change in the underlying mechanism itself, specifically hinting at a decreased energetic efficiency in facilitating the decomposition of glass oxide, subsequently leading to the reduced generation of nanopores and, likely, molecular oxygen. Indeed, the generation of nanogratings inherently involves the formation of molecular oxygen [17], a process that appears to be more difficult in densified silica. Recent studies [41] have evidenced a diminished capacity to generate molecular oxygen in densified silica during electron irradiation. This aligns well with our findings; the challenging nature of initiating molecular oxygen formation and creating associated defects, such as Frenkel defects, seems to inhibit the successful generation of nanogratings in densified samples.

As suggested above, glass free volume could serve as a primary seed for the nanocavitation process. The rapid transition from nanoplasma hotspots into nanopores is driven by swift temperature transfer to phonons and local thermal expansion, which effectively imprints oblate nanopores [18]. These nanopores’ shapes and orientations are largely influenced by the incident light’s polarization, supporting a plasma-mediated over a thermo-mediated nanocavitation process. Recent findings [54] have validated this by demonstrating spherical nanopore formation under circular polarization. For nanocavitation, primary conditions are required: a quick process that surpasses thermal diffusion and acoustic wave relaxation and a significant localized strain coupled with a pressure drop potentially due to local electrostriction [55].

## 5. Conclusions

By employing fs-laser writing on silica glass samples with varied densities, we demonstrated that the tendency for nanograting formation is inversely related to the material’s densification. Densified samples exhibited significantly lower retardance values and slopes, revealing a diminished efficiency in nanograting generation. This provides novel insights into the role of glass free volume as a crucial precursor of nanograting formation. Void-related free volume may serve as an ideal seed owing to its size and lower refractive index, thereby facilitating the formation of nanoplasma hotspots and subsequent nanogratings that consistently align perpendicularly to light polarization. Our suggested mechanism may coherently explain why silica glass or GeO_2_, which naturally possesses a high free volume, facilitates nanograting formation more efficiently than other types of glass. The relationship between free volume and nanograting efficiency could guide the engineering of materials (e.g., nanoporous dedicated materials) with tailored optical properties, paving the way for improved applications in advanced photonic systems.

## Data Availability

Data are contained within the article.

## References

[1] Itoh K., Watanabe W., Nolte S., Schaffer C.B. (2006). Ultrafast Processes for Bulk Modification of Transparent Materials. MRS Bull..

[2] Davis K.M., Miura K., Sugimoto N., Hirao K. (1996). Writing Waveguides in Glass with a Femtosecond Laser. Opt. Lett..

[3] Bricchi E., Klappauf B.G., Kazansky P.G. (2004). Form Birefringence and Negative Index Change Created by Femtosecond Direct Writing in Transparent Materials. Opt. Lett..

[4] Zhang J., Gecevičius M., Beresna M., Kazansky P.G. (2014). Seemingly Unlimited Lifetime Data Storage in Nanostructured Glass. Phys. Rev. Lett..

[5] Rowstron A. (2018). Rethinking Data Storage for the Zettabyte Cloud Era: The Journey from Metal to Glass. Proceedings of the Advanced Photonics 2018 (BGPP, IPR, NP, NOMA, Sensors, Networks, SPPCom, SOF).

[6] Mihailov S., Grobnic D., Hnatovsky C., Walker R., Lu P., Coulas D., Ding H. (2017). Extreme Environment Sensing Using Femtosecond Laser-Inscribed Fiber Bragg Gratings. Sensors.

[7] Wang Y., Cavillon M., Ballato J., Hawkins T., Elsmann T., Rothhardt M., Desmarchelier R., Laffont G., Poumellec B., Lancry M. (2022). 3D Laser Engineering of Molten Core Optical Fibers: Toward a New Generation of Harsh Environment Sensing Devices. Adv. Opt. Mater..

[8] Bellouard Y., Said A., Dugan M., Bado P. (2004). Fabrication of High-Aspect Ratio, Micro-Fluidic Channels and Tunnels Using Femtosecond Laser Pulses and Chemical Etching. Opt. Express.

[9] Hnatovsky C., Taylor R.S., Simova E., Rajeev P.P., Rayner D.M., Bhardwaj V.R., Corkum P.B. (2006). Fabrication of Microchannels in Glass Using Focused Femtosecond Laser Radiation and Selective Chemical Etching. Appl. Phys. A Mater. Sci. Process..

[10] Lu J., Dai Y., Li Q., Zhang Y., Wang C., Pang F., Wang T., Zeng X. (2019). Fiber Nanogratings Induced by Femtosecond Pulse Laser Direct Writing for In-Line Polarizer. Nanoscale.

[11] Beresna M., Gecevičius M., Kazansky P.G., Gertus T. (2011). Radially Polarized Optical Vortex Converter Created by Femtosecond Laser Nanostructuring of Glass. Appl. Phys. Lett..

[12] Beresna M., Gecevičius M., Kazansky P.G. (2014). Ultrafast Laser Direct Writing and Nanostructuring in Transparent Materials. Adv. Opt. Photonics.

[13] Mao S.S., Quéré F., Guizard S., Mao X., Russo R.E., Petite G., Martin P. (2004). Dynamics of Femtosecond Laser Interactions with Dielectrics. Appl. Phys. A Mater. Sci. Process..

[14] Shimotsuma Y., Kazansky P.G., Qiu J., Hirao K. (2003). Self-Organized Nanogratings in Glass Irradiated by Ultrashort Light Pulses. Phys. Rev. Lett..

[15] Rudenko A., Colombier J.-P., Itina T.E. (2016). From Random Inhomogeneities to Periodic Nanostructures Induced in Bulk Silica by Ultrashort Laser. Phys. Rev. B..

[16] Bhardwaj V.R., Simova E., Rajeev P.P., Hnatovsky C., Taylor R.S., Rayner D.M., Corkum P.B. (2006). Optically Produced Arrays of Planar Nanostructures inside Fused Silica. Phys. Rev. Lett..

[17] Lancry M., Poumellec B., Canning J., Cook K., Poulin J.-C., Brisset F. (2013). Ultrafast Nanoporous Silica Formation Driven by Femtosecond Laser Irradiation. Laser Photon. Rev..

[18] Xie Q., Shchedrina N., Cavillon M., Poumellec B., Lancry M. (2023). Nanoscale Investigations of Femtosecond Laser Induced Nanogratings in Optical Glasses. Nanoscale Adv..

[19] Rudenko A., Colombier J.-P., Höhm S., Rosenfeld A., Krüger J., Bonse J., Itina T.E. (2017). Spontaneous Periodic Ordering on the Surface and in the Bulk of Dielectrics Irradiated by Ultrafast Laser: A Shared Electromagnetic Origin. Sci. Rep..

[20] Buschlinger R., Nolte S., Peschel U. (2014). Self-Organized Pattern Formation in Laser-Induced Multiphoton Ionization. Phys. Rev. B Condens. Matter Mater. Phys..

[21] Taylor R., Hnatovsky C., Simova E. (2008). Applications of Femtosecond Laser Induced Self-Organized Planar Nanocracks inside Fused Silica Glass. Laser Photon. Rev..

[22] Lei Y., Shayeganrad G., Wang H., Sakakura M., Yu Y., Wang L., Kliukin D., Skuja L., Svirko Y., Kazansky P.G. (2023). Efficient Ultrafast Laser Writing with Elliptical Polarization. Light Sci. Appl..

[23] Chen S., Xu D., Zhang X., Chen X., Liu Y., Liang T., Yin Z., Jiang S., Yang K., Zeng J. (2022). Reversible Linear-Compression Behavior of Free Volume in a Metallic Glass. Phys. Rev. B..

[24] Liao B., Wu S.-Y., Yang L. (2017). Free Volume: An Indicator of the Glass-Forming Ability in Binary Alloys. AIP Adv..

[25] Zanatta M., Baldi G., Brusa R.S., Egger W., Fontana A., Gilioli E., Mariazzi S., Monaco G., Ravelli L., Sacchetti F. (2014). Structural Evolution and Medium Range Order in Permanently Densified Vitreous SiO_2_. Phys. Rev. Lett..

[26] Ojha P.K., Rath S.K., Sharma S.K., Sudarshan K., Pujari P.K., Chongdar T.K., Gokhale N.M. (2015). Free Volume of Mixed Cation Borosilicate Glass Sealants Elucidated by Positron Annihilation Lifetime Spectroscopy and Its Correlation with Glass Properties. J. Power Sources.

[27] Onodera Y., Kohara S., Salmon P.S., Hirata A., Nishiyama N., Kitani S., Zeidler A., Shiga M., Masuno A., Inoue H. (2020). Structure and Properties of Densified Silica Glass: Characterizing the Order within Disorder. NPG Asia Mater..

[28] Deschamps T., Kassir-Bodon A., Sonneville C., Margueritat J., Martinet C., de Ligny D., Mermet A., Champagnon B. (2013). Permanent Densification of Compressed Silica Glass: A Raman-Density Calibration Curve. J. Phys. Condens. Matter.

[29] Zhang F., Zhang H., Dong G., Qiu J. (2014). Embedded Nanogratings in Germanium Dioxide Glass Induced by Femtosecond Laser Direct Writing. J. Opt. Soc. Am. B.

[30] Yao H., Xie Q., Cavillon M., Dai Y., Lancry M. (2024). Materials Roadmap for Inscription of Nanogratings inside Transparent Dielectrics Using Ultrafast Lasers. Prog. Mater. Sci..

[31] Poe B.T., Romano C., Henderson G. (2004). Raman and XANES Spectroscopy of Permanently Densified Vitreous Silica. J. Non-Cryst. Solids.

[32] Guerette M., Ackerson M.R., Thomas J., Yuan F., Bruce Watson E., Walker D., Huang L. (2015). Structure and Properties of Silica Glass Densified in Cold Compression and Hot Compression. Sci. Rep..

[33] Guerette M., Ackerson M.R., Thomas J., Watson E.B., Huang L. (2018). Thermally Induced Amorphous to Amorphous Transition in Hot-Compressed Silica Glass. J. Chem. Phys..

[34] Martinet C., Kassir-Bodon A., Deschamps T., Cornet A., Le Floch S., Martinez V., Champagnon B. (2015). Permanently Densified SiO_2_ Glasses: A Structural Approach. J. Phys. Condens. Matter.

[35] Lide D.R. (2001). CRC Handbook of Chemistry and Physics.

[36] Tan C.Z., Arndt J., Xie H.S. (1998). Optical Properties of Densified Silica Glasses. Phys. B Condens. Matter.

[37] Güler E., Uğur G., Uğur Ş., Güler M. (2020). A Theoretical Study for the Band Gap Energies of the Most Common Silica Polymorphs. Chin. J. Phys..

[38] Delly J.G. Sénarmont Compensation: How to Accurately Measure Small Relative Retardations (0-1λ). https://www.mccrone.com/mm/measure-small-retardation-senarmont-compensation/.

[39] Xie Q., Cavillon M., Poumellec B., Pugliese D., Janner D., Lancry M. (2022). Application and Validation of a Viscosity Approach to the Existence of Nanogratings in Oxide Glasses. Opt. Mater..

[40] Poumellec B., Lancry M., Chahid-Erraji A., Kazansky P.G. (2011). Modification Thresholds in Femtosecond Laser Processing of Pure Silica: Review of Dependencies on Laser Parameters [Invited]. Opt. Mater. Express.

[41] Ollier N., Reghioua I., Cavani O., Mobasher M., Alessi A., le Floch S., Skuja L. (2023). Probing Densified Silica Glass Structure by Molecular Oxygen and E’ Center Formation under Electron Irradiation. Sci. Rep..

[42] The First Order (Full Wave) Retardation Plate. https://www.olympus-lifescience.com/en/microscope-resource/primer/techniques/polarized/firstorderplate/.

[43] Dai Y., Ye J., Gong M., Ye X., Yan X., Ma G., Qiu J. (2014). Forced Rotation of Nanograting in Glass by Pulse-Front Tilted Femtosecond Laser Direct Writing. Opt. Express.

[44] Rudenko A., Mauclair C., Garrelie F., Stoian R., Colombier J.P. (2019). Light Absorption by Surface Nanoholes and Nanobumps. Appl. Surf. Sci..

[45] Richter S., Jia F., Heinrich M., Döring S., Peschel U., Tünnermann A., Nolte S. (2012). The Role of Self-Trapped Excitons and Defects in the Formation of Nanogratings in Fused Silica. Opt. Lett..

[46] Beresna M., Gecevičius M., Kazansky P.G., Taylor T., Kavokin A.V. (2012). Exciton Mediated Self-Organization in Glass Driven by Ultrashort Light Pulses. Appl. Phys. Lett..

[47] Devine R.A.B. (1994). Macroscopic and Microscopic Effects of Radiation in Amorphous SiO2. Nucl. Instrum. Methods Phys. Res. B.

[48] Cerkauskaite A., Drevinskas R., Rybaltovskii A.O., Kazansky P.G. (2017). Ultrafast Laser-Induced Birefringence in Various Porosity Silica Glasses: From Fused Silica to Aerogel. Opt. Express.

[49] Stopkin S.I., Lipatiev A.S., Fedotov S.S., Lipatieva T.O., Mikhailov Y.V., Lotarev S.V., Sigaev V.N. (2023). Direct Laser Writing of High Retardance Structures in Nanoporous Glass. Proceedings of the International Conference on Advanced Laser Technologies (ALT).

[50] Liu P., Januchta K., Jensen L.R., Bauchy M., Smedskjaer M.M. (2020). Competitive Effects of Free Volume, Rigidity, and Self-adaptivity on Indentation Response of Silicoaluminoborate Glasses. J. Am. Ceram. Soc..

[51] Ojovan M.I. (2008). Viscosity and Glass Transition in Amorphous Oxides. Adv. Condens. Matter Phys..

[52] Xie Q., Cavillon M., Poumellec B., Lancry M. (2023). Upper Temperature Limit for Nanograting Survival in Oxide Glasses: Publisher’s Note. Appl. Opt..

[53] Mobasher M., Lancry M., Lu J., Neuville D., Bellot Gurlet L., Ollier N. (2022). Thermal Relaxation of Silica Phases Densified under Electron Irradiation. J. Non Cryst. Solids.

[54] Sakakura M., Lei Y., Wang L., Yu Y.-H., Kazansky P.G. (2020). Ultralow-Loss Geometric Phase and Polarization Shaping by Ultrafast Laser Writing in Silica Glass. Light Sci. Appl..

[55] Poumellec B., Cavillon M., Lancry M. (2023). Electrostatic Interpretation of Phase Separation Induced by Femtosecond Laser Light in Glass. Crystals.

